# 异基因造血干细胞移植后防治乙型肝炎病毒再激活中国专家共识（2023年版）

**DOI:** 10.3760/cma.j.issn.0253-2727.2023.06.001

**Published:** 2023-06

**Authors:** 中华医学会血液学分会造血干细胞应用学组

异基因造血干细胞移植（allo-HSCT）是治疗血液系统恶性疾病的主要方法，allo-HSCT数量在全球范围内逐年增长。中国是乙型肝炎病毒（HBV）的中度流行地区，流行病学数据显示，我国一般人群的乙型肝炎表面抗原（HBsAg）携带率为5％～6％[1]。2017年至2020年，乙型肝炎在我国的年发病率为1％左右[2]。HBsAg阳性或既往感染HBV的患者在接受强免疫抑制治疗、化疗、单克隆抗体靶向治疗（尤其是抗CD20单克隆抗体）时存在HBV再激活的风险[3]–[5]。而接受allo-HSCT治疗的血液病患者HBV再激活发生风险较其他患者更高[6]–[13]。目前国内仍缺乏allo-HSCT后防治HBV再激活的规范化临床共识或标准。为此，中华医学会血液学分会干细胞应用学组组织有关专家进行了讨论，在回顾大量国内外文献的基础上，结合中国的实际情况，就血液病患者allo-HSCT后防治HBV再激活达成共识，旨在为血液科、造血干细胞移植亚专科及相关医师提供临床指导。

一、定义和流行病学

慢性HBV感染：HBsAg（或）HBV-DNA阳性6个月以上。

既往HBV感染：HBsAg阴性且乙型肝炎核心抗体（HBcAb）阳性[14]。

隐匿性HBV感染（OBI）：肝脏/外周血中存在HBV-DNA而HBsAg阴性。根据HBsAb/HBcAb的状况可分为血清阳性OBI（HBsAb/HBcAb阳性）和血清阴性OBI（所有血清学指标均阴性）[15]。

HBV再激活：慢性HBV感染或既往HBV感染患者在allo-HSCT后出现HBV再次复制，HBV-DNA水平与基线相比显著上升或HBsAg由阴性转为阳性[8]。

HBV再激活的风险依据患者的HBV血清学状况而不同。HBsAg阳性患者接受allo-HSCT具有较高的HBV再激活风险[16]–[17]。在未接受预防性抗病毒治疗的HBsAg阳性患者中，移植后HBV再激活的发生率高达45％～81％[16],[18]–[19]。在未接受预防性抗病毒治疗的既往HBV感染患者中，移植后HBV再激活的发生率为4.3％～40.8％[13],[20]–[24]。HBV还可通过HBsAg阳性供者的造血干细胞传输给allo-HSCT受者[25]。在未行干预情况下，以HBsAg阳性供者进行allo-HSCT，移植后患者HBV相关肝炎的发生率高达48％～55.5％[26]–[27]。调查发现15.3％的HBsAg阴性造血干细胞移植供者存在OBI，其中73.7％的OBI供者同时存在HBsAb阳性[28]。目前没有接受OBI供者allo-HSCT后患者HBV相关肝炎发生率的报道，但已证实OBI患者在接受抗肿瘤化疗或者其他免疫抑制治疗时存在HBV再激活的风险。

二、HBV再激活的发生机制

HBV经母婴、血液和性接触传播进入人体，在体内复制并通过肝脏特异性受体进入肝细胞内，HBV的核酸进入到肝细胞细胞核中，转变成共价闭合环状脱氧核糖核酸（cccDNA）[29]–[30]。HBV感染人体后，机体分别经历免疫耐受期（HBV-DNA在体内复制），免疫清除期（免疫系统清除HBV-DNA和识别杀伤感染HBV的肝细胞）和免疫控制期（血清清除HBV-DNA，cccDNA存在于肝细胞中）[31]–[32]。尽管血清中清除了HBV，HBV的少量cccDNA仍稳定并持续存在于肝细胞核内[33]–[34]。当机体处于免疫抑制状态时，HBV特异性T细胞的细胞毒作用降低，B细胞产生的HBsAb减少，HBV-DNA在体内再次复制[35]–[39]。当机体免疫功能恢复时，病毒引起的免疫应答导致肝细胞损伤和炎性坏死[40]。

三、HBV再激活的诊断和临床评估

（一）诊断标准

HBsAg阳性患者：①HBV-DNA较基线升高≥ 2 log；②移植前血清未检测到HBV-DNA，移植后HBV-DNA≥ 2 log（100）IU/ml；③如果移植前HBV-DNA的基线水平未知，移植后HBV-DNA≥ 4 log（10 000）IU/ml。

既往HBV感染（HBsAg阴性、HBcAb阳性）患者：①移植前HBsAg阴性，移植后HBsAg转为阳性；②移植前血清未检测到HBV-DNA，移植后检测到HBV-DNA[4],[41]–[43]。

肝炎发作（hepatitis flare）定义为丙氨酸转氨酶≥3倍基线水平且>100 U/L。HBV相关肝炎定义为同时存在HBV再激活和肝炎发作。

（二）临床评估

HBV再激活在临床上既可表现为无症状肝炎，也可表现为严重的肝功能衰竭，导致原计划的免疫抑制治疗中断或者延迟，对原发病的治疗产生负面影响[10]。临床需要在一致的HBV再激活定义下根据HBV-DNA病毒载量改变、肝酶和凝血酶原时间国际标准化比值变化、肝炎相关死亡率、对免疫抑制剂减量（或中断）的影响等来评估HBV再激活的严重程度[44]。HBV再激活是一个复杂的临床问题，建议积极与肝病科、消化科、病理科、综合监护室、输血科等开展多学科讨论共同制定临床决策。

（三）鉴别诊断

1. 肝脏移植物抗宿主病（GVHD）：肝脏急、慢性GVHD是移植后常见并发症，临床表现为淤胆性肝损伤为主，胆红素升高伴或不伴肝酶的上升，通常以谷氨酰转肽酶、碱性磷酸酶升高为主。急性肝脏GVHD发生于移植后早期，多伴有皮疹、墨绿色水样便等其他器官的急性GVHD症状；慢性肝脏GVHD发生于移植后晚期，多伴有干眼、口腔溃疡、腹泻、皮疹、关节僵硬等其他器官的慢性GVHD症状；必要时进行肝脏活检，病理可见大量异源性T淋巴细胞浸润伴肝内小胆管的损伤和肝细胞的损伤。

2. 药物性肝损伤：需要根据病史和临床表现，排除肝损害的其他原因后，评估可疑药物应用与肝损害的因果关系。目前常用的是RUCAM量表因果关系评分标准[45]。停用可疑药物或清除体内药物及其代谢产物后肝损害可恢复[46]。移植后应用钙调蛋白抑制剂、三唑类抗真菌药等可能引起肝损伤，需特别关注。

3. 肝窦阻塞综合征（SOS）/肝小静脉闭塞综合征（VOD）：造血干细胞移植后SOS多发生于移植后21 d内，主要由预处理相关肝毒性导致。表现为痛性肝肿大、黄疸、腹水、体重增加（≥5％）、水肿等，实验室检查可见高胆红素血症（总胆红素>34.2 mmol/L或2 mg/dl）、转氨酶升高、难以解释的血小板减少，其诊断主要依赖于临床表现，肝脏超声、CT和MRI等影像学检查可辅助诊断，必要时需行经颈静脉测量肝静脉压力梯度及肝穿刺活检。

4. 移植相关血栓性微血管病（TA-TMA）：TA-TMA是一类以微血管性溶血性贫血、血小板减少、微血栓形成和多器官功能障碍为主要临床表现的HSCT后严重并发症。可依据乳酸脱氢酶（LDH）升高、蛋白尿、高血压、新发的血小板减少（血小板计数<50×10^9^/L或较基线水平下降≥50％）、新发的贫血、溶血、微血管病变证据、终末补体活化（血浆sC5b-9高于正常上限）证据来鉴别。TA-TMA主要累及肾脏，肝脏受累少见。

此外，还需要与细菌或真菌感染、其他病毒性肝炎、非嗜肝病毒感染、毛细血管渗漏综合征等进行鉴别。

四、造血干细胞移植后HBV再激活防治策略

建议移植前所有患者和供者筛查HBV血清学标志物（HBsAg、HBsAb、HBcAb）和HBV-DNA。

（一）造血干细胞移植受者的处理建议

1. 慢性HBV感染患者：移植前至少1周开始接受预防性核苷（酸）类似物（NA）治疗，应选用恩替卡韦（ETV）、富马酸替诺福韦二吡呋酯（TDF）和富马酸丙酚替诺福韦（TAF）等强效、低耐药药物。抗病毒药物至少持续至免疫抑制剂停药后18个月。移植后HBsAg清除且HBsAb持续阳性的患者，可优先考虑停止抗病毒治疗（建议肝病专科会诊）。HBV血清学标志物和HBV-DNA监测：①抗病毒治疗期间每3个月1次；②停止抗病毒治疗后前3个月每月1次、以后每3个月1次；③任何时间出现肝功能损害时（图1）。可优选HBsAb阳性供者。

**图1:**
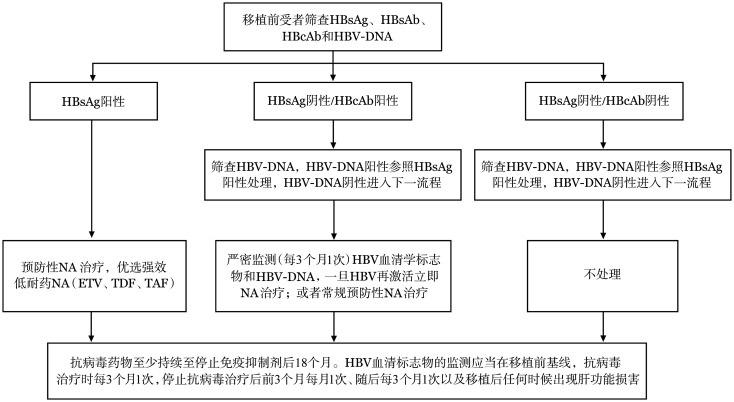
针对异基因造血干细胞移植患者预防乙型肝炎病毒再激活管理流程 注 HBsAg：乙型肝炎表面抗原；HBsAb：乙型肝炎表面抗体；HBcAb：乙型肝炎核心抗体；NA：核苷（酸）类似物；ETV：恩替长韦；TDF：富马酸替诺福韦二吡呋酯；TAF：富马酸丙酚替诺福韦

预防性拉米夫定（LAM）或ETV治疗均能有效降低HBsAg阳性患者allo-HSCT后HBV再激活的发生率[12],[18],[47]–[50]。在接受allo-HSCT的HBsAg阳性患者中，ETV组较LAM组具有更低的HBV再激活发生率[51]。以往慢性乙型肝炎药物治疗方面的研究显示长期使用LAM易发生耐药[6],[52]，且Meta分析显示ETV和TAF对预防慢性HBV感染者免疫抑制治疗后HBV再激活最有效[53]。免疫抑制剂治疗相关指南/共识建议慢性HBV感染患者抗病毒治疗持续至免疫抑制剂停药后6～18个月[41],[52],[54]–[57]。

HBsAb阳性allo-HSCT供者对患者HBV再激活具有保护性作用，供者的HBsAb可降低allo-HSCT患者HBV再激活的风险[12],[58]。HBsAb阳性、阴性供者allo-HSCT患者移植后5年HBV再激活率分别为8.4％、16.3％[23]。此外，HBsAb阳性供者有利于HBsAg阳性患者allo-HSCT后血清HBsAg清除[12],[50],[59]–[60]。

2. 既往HBV感染患者：移植后每3个月1次监测HBV血清学标志物和HBV-DNA，HBV再激活后立即启动NA治疗，或者在移植前常规预防性NA治疗。NA应使用ETV、TDF、TAF。抗病毒药物至少持续至免疫抑制剂停药后18个月。对于移植后HBsAg清除且HBsAb持续阳性患者可优先考虑停止抗病毒治疗（建议肝病专科会诊）。HBV血清学标志物和HBV-DNA监测：①抗病毒期间每3个月1次；②停止抗病毒治疗后前3个月每月1次、后续每3个月1次；③任何时间出现肝功能损害时（图1）。

既往HBV感染患者在接受allo-HSCT时否需要进行预防性抗病毒治疗仍存争议[41]–[43], [52], [54], [56]–[57], [61]。既往研究显示预防性LAM能显著降低HBV感染患者allo-HSCT后HBV再激活发生率[62]–[64]。然而研究显示早期预防性抗病毒治疗不能有效阻止allo-HSCT后晚期HBV再激活[65]–[66]。国内报道HBV再激活为allo-HSCT后晚期并发症，HBV既往感染患者在不进行预防性抗病毒治疗情况下，allo-HSCT后HBV再激活的发生率为4.3％～4.9％[13],[20]。

3. OBI：预防措施同HBsAg阳性者。OBI受者接受包含抗CD20单克隆抗体治疗时，HBV再激活发生率较高（>10％）[15],[67]–[68]。鉴于供者OBI的可能性[28]，输入OBI供者移植物可能会造成受者allo-HSCT后HBV再激活，allo-HSCT供者均应在移植前筛查HBV-DNA。若HBV-DNA阳性，则参照HBsAg阳性供者处理。

4. 患者HBsAg和HBV-DNA阴性，供者HBsAg和（或）HBV-DNA阳性：以下两种综合处理策略均可采用：①患者在移植前1周开始预防性NA治疗；HBsAb阴性患者移植前可接种1～3剂乙肝疫苗；HBsAb阳性（≥10 IU/L）患者移植前可接种1剂乙肝疫苗。②患者以乙型肝炎免疫球蛋白进行被动免疫（移植当天、移植后1个月、移植后2个月），移植后12个月起按照0、1、6个月时间间隔接种3剂乙肝疫苗（图2）。

**图2:**
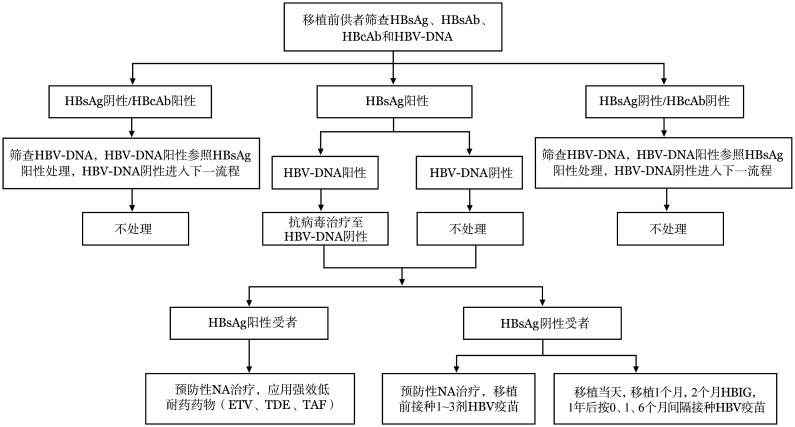
针对异基因造血干细胞移植供者预防乙型肝炎病毒（HBV）再激活管理流程 注 HBsAg：乙型肝炎表面抗原；HBsAb：乙型肝炎表面抗体；HBcAb：乙型肝炎核心抗体；NA：核苷（酸）类似物；ETV：恩替长韦；TDF：富马酸替诺福韦二吡呋酯；TAF：富马酸丙酚替诺福韦；HBIG：乙型肝炎免疫球蛋白

5. 患者HBsAg和HBV-DNA阴性，供者HBsAg阴性、HBcAb阳性：患者移植前可接种乙肝疫苗，移植后密切监测HBV指标。

第五届欧洲白血病感染会议指出，供者为HBsAg和（或）HBV-DNA阳性时，供者和患者均进行抗病毒治疗，同时HBsAg阴性患者进行乙肝疫苗接种可有效阻断HBV传输、减少HBV相关肝炎的发生[26],[69]。国内对检测到HBV-DNA的供者给予抗病毒治疗至HBV-DNA转为阴性；HBsAg阴性患者行乙型肝炎免疫球蛋白被动免疫可达到与HBsAg阴性供者移植相似的移植后HBV相关肝炎发生率[70]。因此，应用接受HBsAg阳性供者的整体处理策略可使HBsAg阳性人群成为allo-HSCT的供者，有助于allo-HSCT在HBV流行地区的开展。

（二）造血干细胞移植供者的处理建议

1. 供者HBV阳性：使用NA治疗至HBV-DNA转为阴性再行造血干细胞采集。

2. 供者HBsAg阳性、HBV-DNA阴性：可直接采集干细胞而无需进行抗病毒治疗。

（三）乙肝疫苗接种的建议

1. 移植前供者、受者HBV血清学标志物均阴性：患者在移植后6个月起可按照0、1、6个月时间间隔接种3剂乙肝疫苗。

2. 患者移植前接种过乙肝疫苗，移植后HBsAb转阴：移植后丢失保护性抗体的患者，可在移植后6～12个月起按照0、1、6个月时间间隔接种3剂乙肝疫苗。

3. 移植后HBsAb未转阳或低滴度阳性：既往HBV感染的患者，移植后常规监测HBsAb滴度，低于保护性作用时可接种乙肝疫苗。如果3剂乙肝疫苗后HBsAb滴度<10 mIU/ml，1～2个月后可追加1剂乙肝疫苗。

乙肝疫苗在移植后患者中有三种作用：①获得对HBV的保护性作用；②使接受HBcAb阳性移植物的患者不发生HBV感染；③在既往HBV感染患者中减低HBV再激活风险[71]。尽管移植前接种了乙肝疫苗，近半数患者在移植后6个月内丢失了对HBV的血清保护作用[72]–[73]，移植后5年超过90％的患者丢失HBsAb的保护性作用[74]。HBsAg阴性患者移植后接种3剂及以上乙肝疫苗，82％～87％的患者能获得保护性HBsAb [75]–[77]，且既往HBV感染患者移植后接种乙肝疫苗能有效降低HBV再激活的发生率[78]–[79]。因此，所有HBsAg阴性患者需在移植后6个月检测HBsAb滴度，必要时重新接种乙肝疫苗。

五、其他需要关注的问题

近年来，细胞免疫治疗和靶向药物得到广泛应用，allo-HSCT患者发生HBV再激活的风险也随之增加[80]–[81]。接受CAR-T治疗慢性HBV感染或既往感染患者的管理可参照《靶向B细胞和浆细增加胞的CAR-T细胞治疗中防治乙型肝炎病毒再激活的中国专家共识》[81]。
